# Step Length Is a Promising Progression Marker in Parkinson’s Disease

**DOI:** 10.3390/s21072292

**Published:** 2021-03-25

**Authors:** Julius Welzel, David Wendtland, Elke Warmerdam, Robbin Romijnders, Morad Elshehabi, Johanna Geritz, Daniela Berg, Clint Hansen, Walter Maetzler

**Affiliations:** 1 Department of Neurology, Kiel University, Arnold-Heller-Straße 3, 24105 Kiel, Germany; David.wendtland@uksh.de (D.W.); e.warmerdam@neurologie.uni-kiel.de (E.W.); r.romijnders@neurologie.uni-kiel.de (R.R.); m.elshehabi@neurologie.uni-kiel.de (M.E.); j.geritz@neurologie.uni-kiel.de (J.G.); d.berg@neurologie.uni-kiel.de (D.B.); c.hansen@neurologie.uni-kiel.de (C.H.); w.maetzler@neurologie.uni-kiel.de (W.M.); 2 Faculty of Engineering, Kiel University, Kaiserstraße 2, 24143 Kiel, Germany

**Keywords:** gait, Parkinson’s disease, inertial measurement unit, movement disorders

## Abstract

Current research on Parkinson’s disease (PD) is increasingly concerned with the identification of objective and specific markers to make reliable statements about the effect of therapy and disease progression. Parameters from inertial measurement units (IMUs) are objective and accurate, and thus an interesting option to be included in the regular assessment of these patients. In this study, 68 patients with PD (PwP) in Hoehn and Yahr (H&Y) stages 1–4 were assessed with two gait tasks—20 m straight walk and circular walk—using IMUs. In an ANCOVA model, we found a significant and large effect of the H&Y scores on step length in both tasks, and only a minor effect on step time. This study provides evidence that from the two potentially most important gait parameters currently accessible with wearable technology under supervised assessment strategies, step length changes substantially over the course of PD, while step time shows surprisingly little change in the progression of PD. These results show the importance of carefully evaluating quantitative gait parameters to make assumptions about disease progression, and the potential of the granular evaluation of symptoms such as gait deficits when monitoring chronic progressive diseases such as PD.

## 1. Introduction

Parkinson’s disease (PD) is a neurodegenerative disease that is typically accompanied by a forward bent upper body, reduced arm swing and short steps with a higher variability in length, width and speed [1]. In clinical supervised assessments, semi-quantitative rating scales such as the Unified PD Rating Scale of the Movement Disorder Society (MDS-UPDRS) are used for the evaluation of disease-related motor symptoms, including gait [2]. Although the MDS-UPDRS shows great agreement with clinicians’ or physicians’ ratings of disease severity, it remains a subjective rating scale that is prone to inaccuracies [3,4]. It is also one of the current gold standards for evaluating axial PD motor symptoms. For example, item 3.8 assesses leg movement, item 3.9 arising from chair, item 3.10 gait and item 3.12 postural stability. All four items can be used to characterize postural instability and gait difficulty score (PIGD) aspects in PD [5]. However, as gait is complex, changes—especially when they are subtle—are very difficult to capture with the naked eye. Moreover, comparison over time to evaluate changes longitudinally can only roughly be estimated when measured semi-quantitatively. Features based on recordings from inertial measurement units (IMUs) might reveal more information about gait impairment and disease progression for patients with PD (PwP).

In the past, quantitative markers from movement tasks (e.g., gait) have shown promising results to monitor progression or evaluate the severity of PD [6,7,8]. For example, a recent study investigating straight and circular walking in PwP with Hoehn and Yahr (H&Y) stages between 1 and 3 and controls over the observation period of 6 years showed that several gait parameters changed in PwP differently from controls [9], basically confirming previous results from a previous study that investigated straight walking in PwP with H&Y stages between 1 and 4 [1]. Exemplary studies investigating the relation between stages of PD and step parameters are shown in Table 1.

Although these papers carefully addressed the relationship between IMU-based parameters and disease progression, a combined set of features explaining biomechanical and clinical mechanisms is rare to find. For example, a recent study has used a model that includes five different domains of gait, but statistical models were restricted to a single quantitative parameter [12]. There have been attempts to derive a composite gait score, evaluating the overall quality of gait in healthy older adults [13,14]. These scores have yet to be related to disease progression in PD. In clinical practice, the impairment of gait is treated using dopaminergic medication which has proven to be very effective, having a positive influence on the previously mentioned gait parameters [15,16]. However, it has previously been shown that disease progression influences step length and step time in straight walking in PwP at different disease stages [1,17]. Here, we extend that research by identifying the relationship between disease stage and gait parameters in 20 m straight walking and circular walking. Our hypothesis was that in PD, disease severity (H&Y 1–4) has a relevant effect on step length and step time in a straight and circular walk paradigm, respectively. For the straight walk, we chose a distance of 20 m, as this distance has been shown to capture sufficient datapoints while not relevantly tiring participants [18]. The consecutive three 360° turns performed in this study were chosen based on clinical experience (sufficient steady-state walking phases for analysis, often dizziness when performing more turns) [7].

Building on this literature, this study aimed to investigate, in PwP with H&Y stages 1–4, the association of step length and step time during straight and circular walking, respectively, with H&Y stage, using a multivariate analysis model. We also investigated the differences between the two walking tasks.

## 2. Materials and Methods

### 2.1. Individuals

Data of 68 PwP from the MODEP (MODelling Epidemiological data to study Parkinson’s disease progression, 45 PwP) and ComOn (Cognitive and Motor Interaction in the Older Population, 23 PwP) study were analysed (see [19,20] for details including inclusion and exclusion criteria). In brief, the MODEP study was a prospective observational study modelling epidemiological data to study Parkinson’s disease progression, and was finished in 2014. The ComOn study is a prospective, exploratory multicentre study exploring cognitive and motor aspects in older patients. It was initiated in 2016 and investigates, among other aspects, correlations between the cognitive and motor parameters of advanced PwP. In the work presented here, data from PwP from the study site in Kiel, Germany were used. For both studies, demographic and clinical parameters, including age, height, and sex, were collected from all subjects. The MDS-UPDRS-III score was determined immediately before each gait analysis [2]. In addition, the Levodopa equivalent daily dose (LEDD) was calculated based on the medication at examination [21]. Cognitive function was assessed using the Montreal Cognitive Assessment (MoCA) [22]. A postural instability and gait difficulty (PIGD) score was derived from the four MDS-UPDRS-III items: arising from a chair, gait, postural stability and posture [5]. Presence of freezing of gait (FOG) was determined with the MDS-UPDRS-III freezing of gait item. Presence of depressive symptoms was considered if the Beck Depression Inventory (BDI-II, performed in the MODEP study) score was >14 points [23] and if the Depression-im-Alter-Skala (DIA-S, performed in the ComOn study) score was >4 points [24].

### 2.2. Ethics

The ethical committees of the Medical Faculty of Tuebingen (MODEP, 46/2010) and the Medical Faculty of Kiel (ComOn, D427/17) approved the study protocols, and written informed consent was obtained from all participants.

### 2.3. Quantitative Data

For quantitative data collection during the walking tasks, the CE-certified IMUs Dynaport^®^ (McRoberts, The Hague, Netherlands, for the MODEP participants [19]) and Rehagait^®^ (Hasomed GmbH, Magdeburg, Germany, for the ComOn participants [25]) were used. The IMU in each study was located at the lower back, in the area of L5. Each IMU contains three sensors with three axes of motion: an acceleration sensor for linear acceleration, a gyroscope for angular velocity and a magnetometer. Raw data were recorded at a frequency of 100 Hz. The IMUs in both studies were synchronised via Bluetooth. The assessment was started and stopped by the investigator on a tablet. The application on the tablet included all tasks of the standardised gait protocol. The protocols of both studies were identical in terms of length, sequence of gait tasks and effort level for the patients. Raw data from the sensors were transferred directly to the tablet via Bluetooth and stored there. The following two gait tasks were selected for the analyses of this work:

1. Walking a 20 m track from a standing position on a wide (>2 m) corridor without obstacles [26]. The subjects walked at a self-selected walking speed. The exercise was completed when the participant had completely crossed the finish line.
2. Circular walking around an area with diameter of 1.20 m three times at a self-selected speed, which corresponds to a walking distance of 11.3 m [7]. The start was carried out from a standing position initiated by the instructions of the examiner and the exercise was performed once clockwise (starting with the right leg) and once anticlockwise (starting with the left leg) [19].

**Figure 1:**
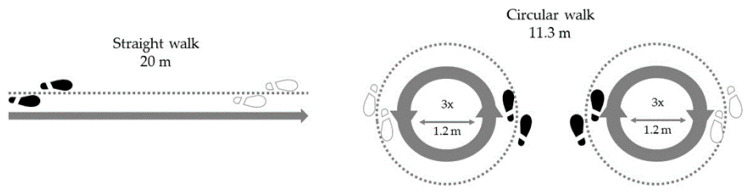
Overview of the experimental setting.

Figure 1 gives an overview of the two tasks performed by the PwP. From these two tasks, step length and step time were derived using a validated algorithm [27].

### 2.4. Statistics

Data were analysed with MATLAB (MathWorks, 2020a) and JASP (JASP Team, 2020. Version 0.13.1). Demographic and quantitative gait parameters were checked for normality of distribution using a Shapiro–Wilk test. Values exceeding the 1.5 interquartile range were defined as outliers and excluded from the analysis. The effect of H&Y stages on quantitative gait parameters were assessed using an analysis of covariance (ANCOVA) controlling for age and height. ANCOVAs were performed for each task (20 m straight walk and circular walk) and each parameter (step length and step time) independently. Post hoc tests with bootstrapped confidence intervals (CIs) were used to compare each of the parameters between H&Y stages. A stepwise multiple linear regression model was used to predict the total task time for the straight and circular walking paradigm from the parameters step length, step time, age, and height. For this model, multicollinearity between the parameters was assessed using the variance inflation factor (VIF).

## 3. Results

### 3.1. Demographic and Clinical Parameters

The demographic and clinical parameters of individuals are summarized in Table 2. The investigated cohorts showed a normal distribution in all clinical parameters, except for age in H&Y stage 1 and MDS-UPDRS-III in H&Y stage 2.

### 3.2. Straight Walk

**Figure 2:**
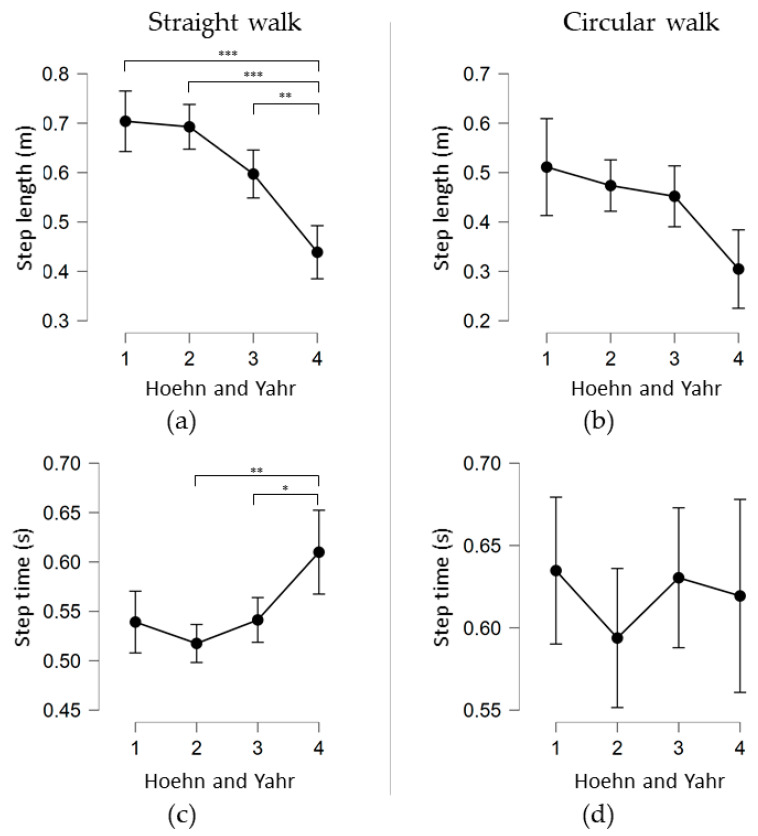
Mean and CI (95%) for each gait parameter separated for each Hoehn and Yahr stage. Statistically significant post hoc comparisons are highlighted (* *p* ≤ 0.05, ** *p* ≤ 0.01, *** *p* ≤ 0.001). (**a**) Step length per Hoehn and Yahr stage for the 20 m straight walk, (**b**) Step length per Hoehn and Yahr stage for the circular walk, (**c**) Step time per Hoehn and Yahr stage for the 20 m straight walk, (**d**) Step time per Hoehn and Yahr stage for the circular walk

There was a significant effect of H&Y stage on step length after controlling for height and age (Figure 2a). Post hoc testing using bootstrapped CIs and Bonferroni corrections revealed that PwP in H&Y stage 4 showed significantly shorter step lengths than PwP in H&Y stages 1 (*p* < 0.001), 2 (*p* < 0.001) and 3 (*p* = 0.002). There were no significant differences in step length between any of the groups H&Y stage 1–3. Additionally, the covariates height and age showed a significant effect on the step length (F (1,55) = 6.252, *p* = 0.015 and F (1,55) = 4.902, *p* = 0.031).

For step time there was again a significant effect of H&Y stage after controlling for height and age (F(3,56) = 5.235, *p* = 0.003; Figure 2b). Post hoc analysis revealed that PwP in H&Y stage 4 showed significantly longer step times than PwP in H&Y stages 2 (*p* = 0.002) and 3 (*p* = 0.016). No significant differences in step time were found between any other H&Y stages.

### 3.3. Circular Walk

For circular walking there was a significant effect of H&Y stage on step length after controlling for height and age (F(3,57) = 2.902, *p* = 0.042; Figure 2c). Post hoc testing using bootstrapped CIs and Bonferroni corrections revealed no significant differences between any of the groups. The covariate age showed a significant effect on the step length during circular walking.

For step time there was no significant effect of H&Y stage after controlling for height and age (F(3,51) = 0.864, *p* = 0.466; Figure 2d). Post hoc analyses showed no significant differences between any of the groups regarding step time.

### 3.4. Total Task Time

The multiple linear regression using stepwise data entry revealed two significant models for the straight walk task. Step length alone (F(1,54) = 27.5, *p* = < 0.001) or in combination with step time (F(2,53) = 17.9, *p* = < 0.001) could significantly predict total task duration. Step length alone explained most of the variance (*R*^2^ = 0.34). When adding step time to the regression model, the change in R^2^ was 0.07. The VIF in the model including step length and step time was 1.19, suggesting a small amount of collinearity between the variables. Similar results were observed for circular walking. Step length alone (F(1,54) = 30.3, *p* < 0.001) or in combination with age (F(2,53) = 22.1, *p* < 0.001) significantly predicted total task duration. Multiple linear regression using stepwise data entry revealed two significant models for the circular walk task. Step length alone (*R*^2^ = 0.36) or in combination with age (*R*^2^ = 0.46) explained most of any predictors in variance. The VIF in the model including both parameters was 1.2, suggesting a small amount of collinearity between the variables.

## 4. Discussion

The present study investigated gait parameters over the entire range of mobile PwP (i.e., H&Y 1–4) by combining the data from two prospective observational studies in PwP [7,20,26].

For straight walks at self-selected speed, step length emerged as a potentially promising marker for the determination of disease progression at any stage, although the differences were most pronounced between H&Y stages 3 and 4. These results confirm the findings by Hatanaka [1], the only other study to our knowledge assessing step length in relation to the same H&Y stages. The only difference in the study protocols of the studies was the walking distance (10 m in the previous study, 20 m in this study). In the straight walking task in the present study, step time increased significantly for PwP in H&Y stage 4 compared to H&Y stages 2 and 3, but not compared to H&Y stage 1. Considering also the results derived from the regression analyses including step length, step time, height and age as variables, it therefore seems that step time also changes in the course of PD. However, this change seems to be smaller and less reliable than the changes of step length. In fact, step length was the parameter which best predicted total task time and explained most of its variance in straight and circular walking total task time. These results reflect our clinical experience well. Our results are also in agreement with the above-mentioned study [1], as the authors did not find a significant effect of all H&Y stages on step times. Additionally, in circular walking, there was no effect of H&Y stage on step time. We only found a small although significant influence of the H&Y stages on step length. Circular walking might thus reflect different motor and cognitive processes than straight walking [28], and may therefore be less suitable as a task for determining progression in PD.

This study faces some limitations. First, whereas the MODEP participants were mainly assessed in a medication-off state, a relatively high proportion of the more advanced participants from the ComOn cohort had to be on medication to successfully perform the assessment (see also Table 2, which also shows a slightly better MDS-UPDRS-III score in this group compared to the H&Y 3 group). However, our results argue against a relevant influence of this aspect on the observation presented here, as we still saw an overall decrease in step length performance for the H&Y stage 4, suggesting that the deficit may be rather underestimated in this H&Y stage. Second, this study provides data from a cross-sectional protocol, and our results should therefore be confirmed in longitudinal studies, including potential confounders such as cognitive and emotional function. Additionally, FOG, which is most present in H&Y stage 4, can theoretically have an effect on step length and step time. However, although we cannot definitely exclude freezing episodes in our dataset, we did not observe any obvious freezing episodes during the assessments. This is not surprising for this type of assessment and analysis (only continuous straight walking phases and continuous curved walking phases, respectively, on firm ground—see also Figure 1). Third, PD is a heterogeneous disease, and it is possible that we did not account for this aspect comprehensively due to, for example, small sample size. Fourth, the changes in gait parameters might not follow a linear trend between the H&Y stages, but rather change more severely as the disease progresses. Fifth, the regression analysis did not focus on the explanation of H&Y stages but on gait velocity. We would like to point out here that it was not our aim to best fit the data, but rather understand how each of the included parameters influence the total task time. Even though step length and step time are not fully independent, respecting the collinearity measure it seemed reasonable to include both parameters in the analysis at the same time. Finally, including more diverse walking conditions (e.g., different slopes, uneven surfaces) could potentially provide even higher ecological validity to the findings.

Despite these limitations, we still feel that our pilot results could motivate further studies that especially focus on the accurate assessment of different walking parameters for the monitoring, and finally treatment responsivity, of these parameters in PD.

## 5. Conclusions

Our prospective cross-sectional evaluation of step length and step time in PD over a large spectrum of disease severity, using a supervised assessment, suggests that (i) a straight walk paradigm reflects gait changes over the course of PD better than a circular walking protocol; (ii) step length shows a clear and continuous decline in the course of the disease, whereas step time seems to reflect disease severity less reliably; and (iii) these changes are most pronounced in advanced disease changes (i.e., between H&Y stages 3 and 4). Next steps to further evaluate the value of these parameters and assessment strategies are to measure longitudinal cohorts and implement these measures in clinical trials.

## Figures and Tables

**Table 1:** Exemplary studies on gait characteristics associated with the stages of Parkinson’s disease.

Author	Year	Cohort	H&Y Stage	Task	Main Findings
Herman et al. [10]	2014	31 PwP-PIGD, 32 PwP-TD, cross-sectional	1–4	30 m straight walk	Slower gait speed, shorter strides, less smoothness, and excessive instability in PwP-PIGD compared to PwP-TD. No association between step length and H&Y stages investigated.
Curtze et al. [5]	2015	104 PwP cross-sectional	2–4	7 m straight walk with turns	Levodopa intake improves arm swing range/velocity, gait velocity and stride length. No association between step length and H&Y stages investigated.
Hatanaka et al., [1]	2016	124 PwP cross-sectional	1–4	10 m straight walk	Decreased velocity and step length with increasing H&Y
Bayle et al. [11]	2016	39 PwP cross-sectional	2–4	5 m straight walk with turns	The contribution of step length and cadence to increased ambulation speed is age-invariant but a marker of PD. No association between step length and H&Y stages investigated.
Del Din et al. [12]	2019	16 PD converter in a cohort of 696 healthy controls	-	20 m straight walk	Higher step time variability and asymmetry of gait characteristics are associated with a shorter time to PD diagnosis. No association between step length and H&Y stages investigated.
Micò-Amigo et al. [7]	2019	27 PwP longitudinal	0–4	1.2 m circular walk (×3)	Number of steps, total task time, stride time variability, and stride regularity are potential PD progression markers. No association between step length and H&Y stages investigated.
Wilson et al. [9]	2020	109 PwP longitudinal, 130 HC	1–3	25 m oval circuit	Increased variability of swing time, step time and step width, and reduced swing time asymmetry specific to PD when compared to healthy older adults. No association between step length and H&Y stages investigated.

Papers were selected from a PubMed search (January 2012–December 2020) using the keywords step length, Parkinson, progression. HC: healthy controls; H&Y, Hoehn and Yahr; PD, Parkinson’s disease; PIGD, postural instability and gait difficulty score; PwP, people with PD; TD: tremor dominant.

**Table 2:** Demographic and clinical data (mean ± standard deviation) split by H&Y stage.

H&Y Stage	1	2	3	4
N (female)	11 (5)	26 (12)	17 (9)	14 (8)
Age (years)	62 ± 10	62 ± 9	69 ± 5	76 ± 6
Height (m)	1.74 ± 0.11	1.74 ± 0.08	1.70 ± 0.10	1.70 ± 0.10
MDS-UPDRS-III (0–132)	13 ± 7	29 ± 11	34 ± 14	30 ± 13
LEDD (mg/d)	301 ± 187	368 ± 201	416 ± 282	608 ± 320
On/Off (%)	37/63	32/68	44/56	92/8
MoCA (0–30)	25.8 ± 3.0	26.3 ± 3.1	25.1 ± 3.5	23.7 ± 3.1
PIGD (0–16)	1.3 ± 1.0	2.4 ± 2.6	5.4 ± 1.9	8.5 ± 2.0
Freezing (%)	0	0	18	54
Depressive symptoms (%)	20	28	20	27

H&Y: Hoehn and Yahr; LEDD: Levodopa equivalent daily dose; MDS-UPDRS-III: Movement Disorder Society-revised version of the Unified Parkinson’s Disease Rating Scale, motor part; MoCA: Montreal Cognitive Assessment; PIGD: postural instability and gait difficulty.

## Data Availability

Data available on request due to restrictions e.g., privacy or ethical.
